# Improved production of Daptomycin in an airlift bioreactor by morphologically modified and immobilized cells of *Streptomyces roseosporus*

**DOI:** 10.1186/s13568-016-0274-0

**Published:** 2016-10-22

**Authors:** Ipsita Chakravarty, Subir Kundu

**Affiliations:** School of Biochemical Engineering, Indian Institute of Technology, Banaras Hindu University, Varanasi, 221005 India

**Keywords:** Antibiotics, Drug resistance, Morphological variation, Immobilization, Daptomycin

## Abstract

The increased threat of drug resistance has challenged the existence of several conventional and non-conventional antibiotics in the recent times. Daptomycin is a novel cyclic-lipopeptide antibiotic produced by *Streptomyces roseosporus* that has progressed as a significant anti-MRSA (methicillin-resistant *Staphylococcus aureus*) antibiotic. But, the economic practicality of this highly valued secondary metabolite is deterred by its poor production and tedious processing methodology. The present study aims at strategic improvement of Daptomycin production through morphological variations of *S. roseosporus* cells. Free cells, pelletized cells and immobilized cells on ultra porous refractory brick and silk sachets were investigated for the production of Daptomycin in a lab-scale 2.0 l air-lift bioreactor. The effect(s) of nitrogen source, inoculum size and oxygen stress were analyzed for pellet formation of *S. roseosporus.* Interestingly, free cells produced 750 mg/l of Daptomycin in a single batch. But, the three phase broth viscosity increased due to vigorous growth of free cells which hampered the oxygen transfer rate. The cell degeneration over the time deterred pellet reusability. 1430 mg/l Daptomycin was produced using pellets for 2 batches. On the contrary, mechanical stability, buoyancy and reusability of refractory bricks and silk sachets were beneficial. Daptomycin production was recorded for 6–8 batches. Immobilized cells on refractory bricks and silk sachets led to 4895 mg/l and 3623 mg/l Daptomycin production respectively. Cell immobilization improved the three phase broth rheology and hence, the hydrodynamics within the reactor. Therefore, whole-cell immobilization could contribute to the ameliorated production of this life-saving drug.

## Introduction

The health care sector is apprehensive and intimidated about the issue of drug resistance (Fluit et al. 2001). Researchers around the world are engaged with metabolic engineering and genetic improvement of several life-saving drugs that can combat this issue. There is also an urge to improve their production to reduce the cost of its industrial applicability. Therefore, availing it to a larger section of the society. Daptomycin is such a novel cyclic lipopeptide antibiotic produced by *Streptomyces roseosporus*, which works effectively against methicillin-resistant *Staphylococcus aureus* and vancomycin-resistant *Enterococci* due to its unique mechanism of action. This antibiotic has got the FDA approval only in 2003 for the treatment of patients with complicated skin and skin structure infections, right-sided endocarditis and bacteremia (Eisenstein et al. 2010; Chakravarty et al. 2015). The filamentous nature of *S. roseosporus* affects the rheology of the fermentation broth. This hampers its production to a great extent. Though, researchers have attempted to increase the yield through genetic engineering and metabolic flux analysis (Huang et al. 2012).There have been very few attempts towards the process strategies for improved production of Daptomycin. The physiological state and morphological differentiation of filamentous microorganisms can be related to changes in growth conditions during submerged cultivations (Kundu et al. 2000). Morphological features have been correlated with the production of secondary metabolites at several instances (O’Cleirigh et al. 2005; Papagianni and Mattey 2006). Improved antimicrobials’ production was observed through pellet formation (Choi et al. 2000). Also, whole- cell immobilization is a useful processing strategy to instantiate the potential of bioprocesses. Previous reports have contributed to explore how the phenomenon of immobilization affects growth and metabolite production (Kundu et al. 1992; Mahapatra et al. 2002). Higher cell concentration can be maintained by immobilization methods which enhance the productivity of the secondary metabolite as it is a non-growth associated product. Prolonged reusability is also an advantage of whole-cell immobilization (Srivastava and Kundu 1998; Mishra et al. 2005). The objective of the present work was to evaluate the production of Daptomycin through improved broth rheology and controlled hydrodynamics. Therefore, we have attempted some alternative strategies to improve antibiotic production and have tried to study the pattern of oxygen mass transfer using an air-lift bioreactor with different modes of cell growth.

## Materials and methods

### Strain

*Streptomyces roseosporus* NBRC 12910 (Chiba, Japan) was used for these studies.

### Medium and culture conditions

*Streptomyces roseosporus* was cultivated and maintained at 30 °C and 200 rpm in culture broth medium containing (g/l): malt extract 3, glucose 10, yeast 3 and peptone 5 at pH 6.5. Fermentation was carried out in medium containing (g/l) Dextrin 30, Glucose 10, Soyabean flour 20, Fe (NH_4_)_2_ SO4 0.6, KH_2_PO_4_ 0.2, and pH 7 and incubated at 30 °C for 6 days. Cofactors were added to the sterile culture medium after aseptic filtration. Eight cofactors were added which included nicotinic acid(4 mg/l), riboflavin(0.5 mg/l), heme(9 mg/l), thiamine(0.4 mg/l), biotin(0.1 mg/l), cyanocobalamin(0.04 mg/l), tetrahydrofolic acid(6 mg/l) and pyridoxal 5-phosphate(0.4 mg/l) (Yu et al. 2011). As for the airlift bioreactor, the pH was automatically controlled at 7.0 with 8 M NaOH solution and the aeration speed of 1 v/v/m for 6 days. N-decanoic acid was fed at 48 h after inoculation (0.2 g/l). All experiments were done in triplicate.

### Morphological variations of *S. roseosporus*

The growth conditions were altered for pelletization the cells. The morphological variation of *S. roseosporus* was carried out by changing the inoculum size, the nitrogen source and the aeration rate in the fermentation medium. Morphological changes during the fermentation were observed. Suspended pellets were imaged and pellet diameter was calculated, assuming that the pellets were perfectly spherical. Pellet size distribution was calculated by averaging 20–50 pellets. Both visual observations and image processing tools were taken into consideration.

### Immobilization of *S. roseosporus* on refractory brick flakes and silk sachets

**Fig. 1:**
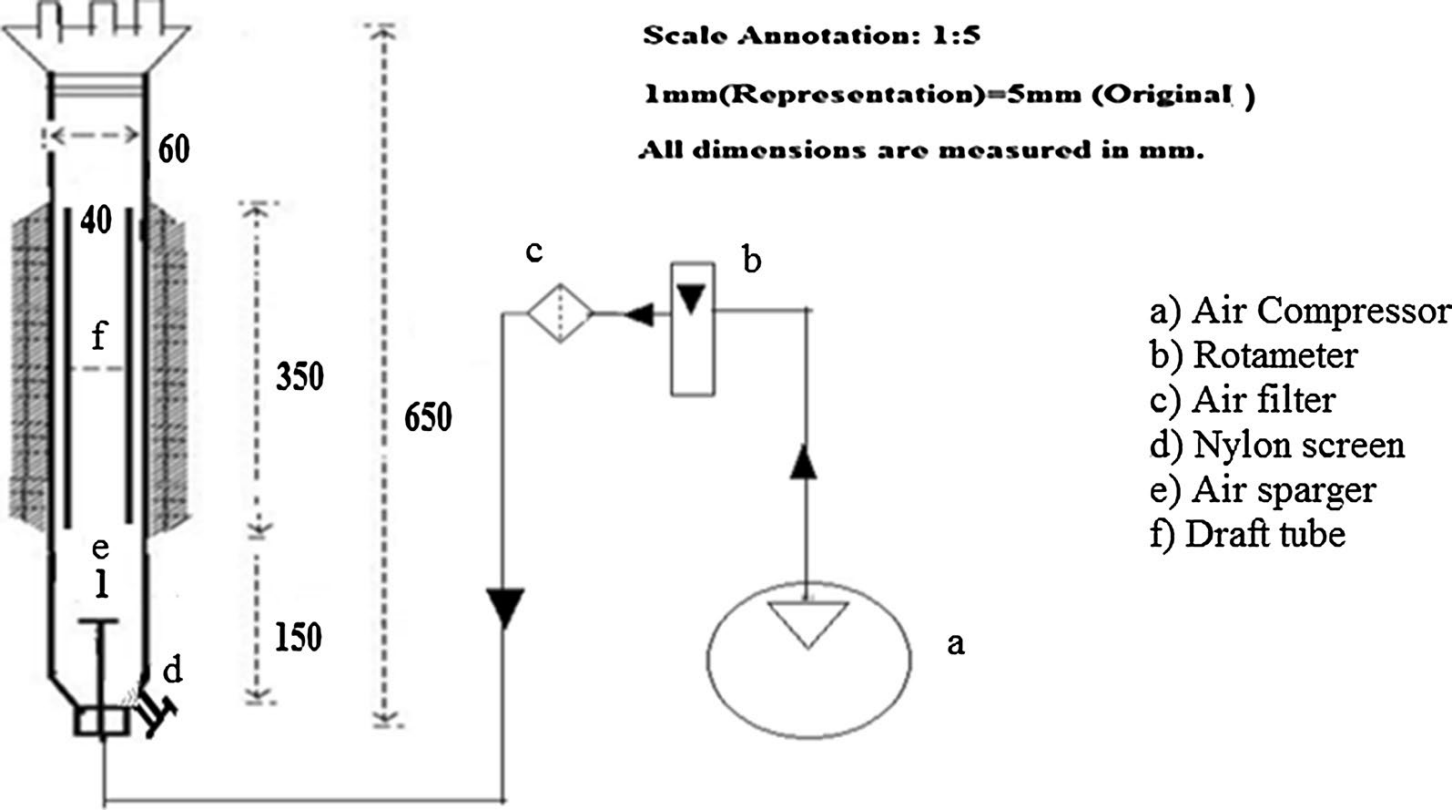
Schematic representation of the airlift bioreactor

Refractory bricks were mechanically crushed and sieved uniformly to particle size of 5 mm. These were pretreated by boiling in water for 20 min at 80 °C and washed with distilled water. They were placed in methanol for 3 h. Then, washed again with distilled water. For the immobilization of *S. roseosporus* on the support matrices, 2.0 g of pretreated carrier were placed in a 250 ml Erlenmeyer flask containing seed medium. After sterilization (121 °C and 15 lbs/in^2^ pressure for 20 min), the flasks were inoculated with 0.5 ml of homogenized mycelia (0.15 dry cell weight of mycelia g/l) under sterile conditions. An equal weight of silk sachets (4 cm × 2 cm) was sterilized, and a concentrated seed culture was put into each silk sachet aseptically. *S. roseosporus* is found to grow on carrier after 5 days of incubation. The growth medium was then removed and the immobilized matrices were thoroughly washed with distilled water under sterile conditions. Experiments were carried out for Daptomycin production by transferring the immobilized matrices into the production medium under sterile conditions. They were loaded into an indigenously developed, presterilized airlift bioreactor containing fermentation media as depicted in Fig. 1 and the dimensions are described in Table 1.

**Table 1:** Table showing the dimensions of the airlift bioreactor

Parameters	Volumetric capacity of reactor	Column diameter	Column height	Ungassed liquid height	Draft tube height	Draft tube diameter	Height of draft tube above the fermenter base	Single sparger hole diameter
Dimensions	2.0 l	60 mm	650 mm	150 mm	350 mm	40 mm	80 mm	1 mm

### Cell growth and product estimation

10 mL broth sample was centrifuged and the supernatant was discarded. Pellets were washed thrice with distilled water. The centrifuged biomass was transferred to a pre-weighed aluminium cup kept at 85 °C for 24 h until a constant weigh was achieved. The dry cell weight was determined by subtracting the weight of aluminium cup from the previous weight. At specified intervals, Daptomycin was analyzed using disk diffusion method using *M. luteus* as the assay organism and confirmed by HPLC.

### Cell leakage estimation

The free cells and cells leaked from the support matrix were collected by centrifugation at 10,000 rpm for 20 min and dried at 90 °C. The initial weight of each support matrix was determined by drying specified quantity to a constant weight. The support matrices with cells were carefully washed with distilled water and dried again. The difference between the weights of the support matrices before and after cell adsorption is considered to be the weight of adsorbed cells (D’souza et al. 1986).

### Evaluation of the rheology and hydrodynamics of fermentation broth

The consequences of the stress conditions subjected in case of different morphological forms were assessed by the rheological properties which were measured using the Brookfield viscometer at different time intervals. The volumetric oxygen transfer coefficient was estimated by the dynamic gassing-out technique. Air flow rate was maintained at 1 vvm (Ruchti et al. 1981).

### Reusability of immobilized and pelletized cells

The cell pellets and the immobilized cells were collected at the 50 micron nylon mesh screen at the sampling port and replenished with fresh fermentation medium for further batches.

### Microscopic analysis of the pelletized and the immobilized cells

Scanning electron microscopy (SEM) was carried out for immobilized cells on refractory bricks to determine the morphological alterations and distribution of cells in the matrices during immobilization. The specimens were chemically fixed and examined in Zeiss scanning electron microscope at 20 kV. All images were digitally recorded. Pelletized cells and silk sachets containing *S. roseosporus* were observed by electron microscopy.

## Results

### Preliminary screening of support matrices

**Fig. 2:**
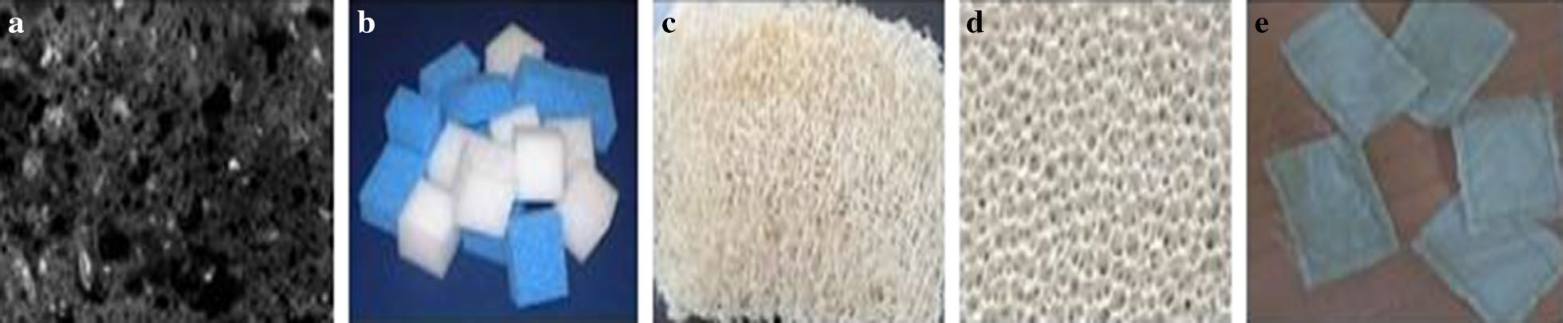
Different immobilization materials screened. **a** Ultra porous refractory brick, **b** polyurethane foam, **c** loofah sponge, **d** ceramic foam, **e** silk sachets

The significance of whole cell immobilization was evaluated by screening five different conventional and non-conventional support matrices viz., ultra porous refractory brick flakes, silk sachets, polyurethane foam, loofah sponge and ceramic foam as shown in Fig. 2. The reusability, retention capacity, immobilization time, mechanical stability and economic viability prompted the use of refractory bricks and silk sachets. Since, airlift bioreactor was used, buoyancy and repeated utility of the matrix was taken into regard.

### Effect of nitrogen sources, inoculum size and aeration rate on pellet formation

**Fig. 3:**
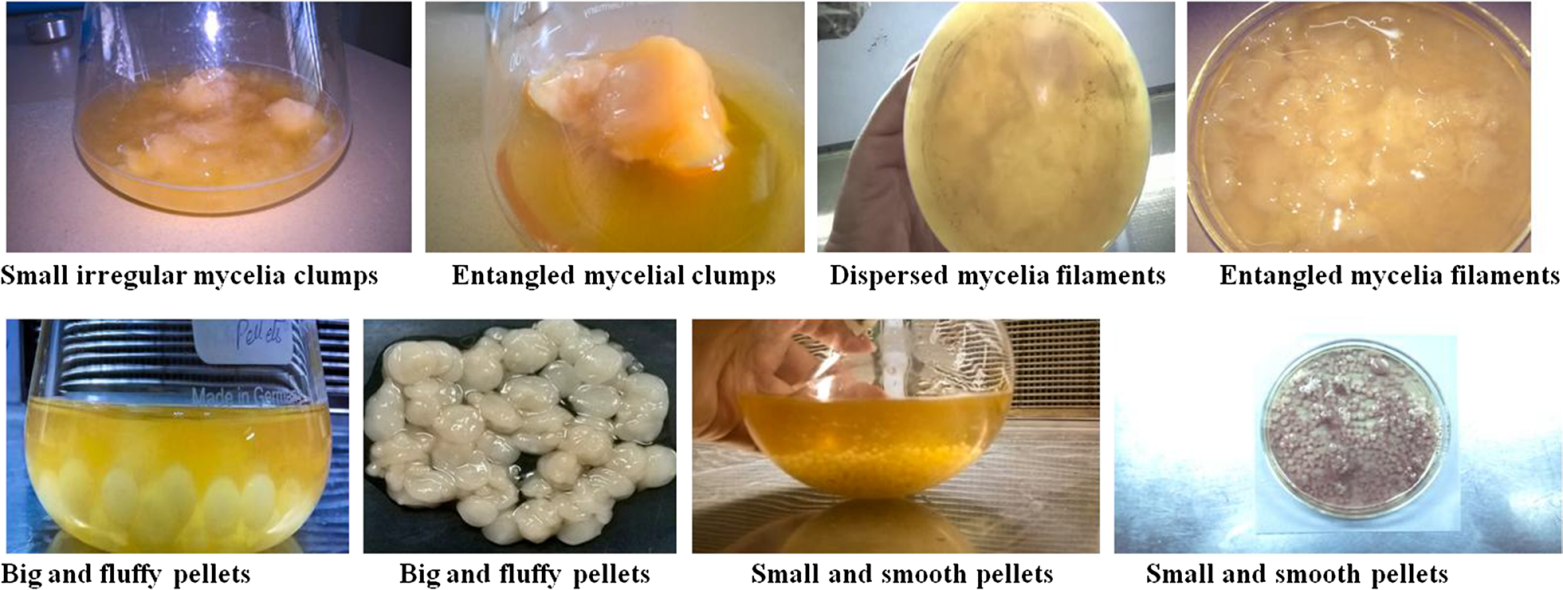
Morphological variations of *S. roseosporus* with different growth conditions

The morphological variation of *S. roseosporus* was carried out by changing the inoculum size, the nitrogen source and reducing aeration rate in the fermentation medium as shown in Table 2. Several visual differences were observed in terms of the branching of hyphae and hyphal arrangement (Fig. 3).

**Table 2:** Table showing the optimization of various conditions for pellet formation

Air flow rate = 0.70 vvm		
Nitrogen source	Inoculum size	
	4 %	5 %
Soyabean meal	Entangled mycelia filaments	Dispersed mycelia filaments
Peptone	Small and smooth pellets	Small irregular mycelia clumps
Yeast extract	Big and fluffy pellets	Entangled mycelial clumps

### Correlation of cell morphology and Daptomycin production

**Fig. 4:**
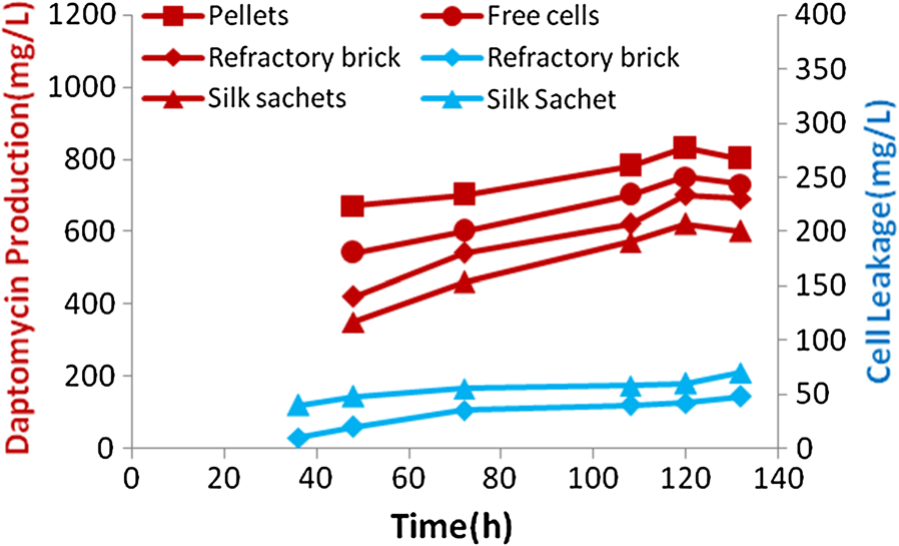
Profiles for Daptomycin production using different mode of cells and cell leakage-free cells, pelletized cells, immobilized cells on refractory brick and immobilized cells on silk sachets

The production profile of immobilized cells revealed that the antibiotic production started at 48 h of fermentation with immobilized cells on refractory bricks and reached maximum level (700 mg/l) by 132 h. On further incubation, no improvement in antibiotic production was observed. In case of free cell fermentation, the maximum antibiotic production (750 mg/l) was observed by 132 h as shown in Fig. 4. The Daptomycin production profile (Fig. 4) with immobilized cells on silk sachets indicate a progress in cell mass and antibiotic titre up to 132 h. The maximum antibiotic yield with immobilized cells on silk sachets reached 620 mg/l. Small and fluffy cell pellets were utilized for Daptomycin production. The growth and Daptomycin production profiles with pellets indicate a progress in cell mass and antibiotic production up to 132 h. The maximum antibiotic yield with pellets reached 810 mg/l.

### Reusability of cells pelletized and immobilized cells

**Fig. 5:**
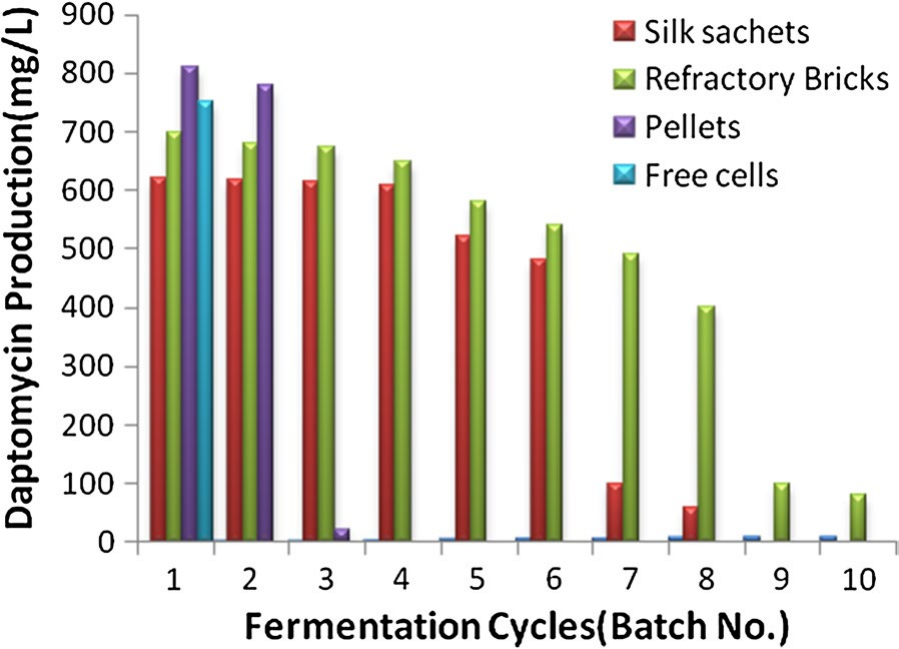
Repeated batch fermentation with *S. roseosporus* cells

Repeated batch fermentation process was carried out for Daptomycin by *S. roseosporus* cells immobilized on refractory brick and silk sachets as shown in Fig. 5. These modes were found to be superior due to low cell leakage and stable for repeated use. Significant process engineering advantages are evident from immobilized cells in repeated batch operations. Reusability of immobilized cells was achieved by aseptic removal of fermentation medium and replacement with afresh medium for *S. roseosporus* production. Daptomycin production was studied up to ten reuse cycles. The fermentation was continued for several batches until the carrier material disintegrated. There was an increase in Daptomycin production up to eighth cycle for refractory brick immobilized cells and later a gradual decrease in antibiotic production was noticed. Similarly, for the silk sachets, the production could be repeated for six cycles.

### Microscopic view of different cell morphology

**Fig. 6:**
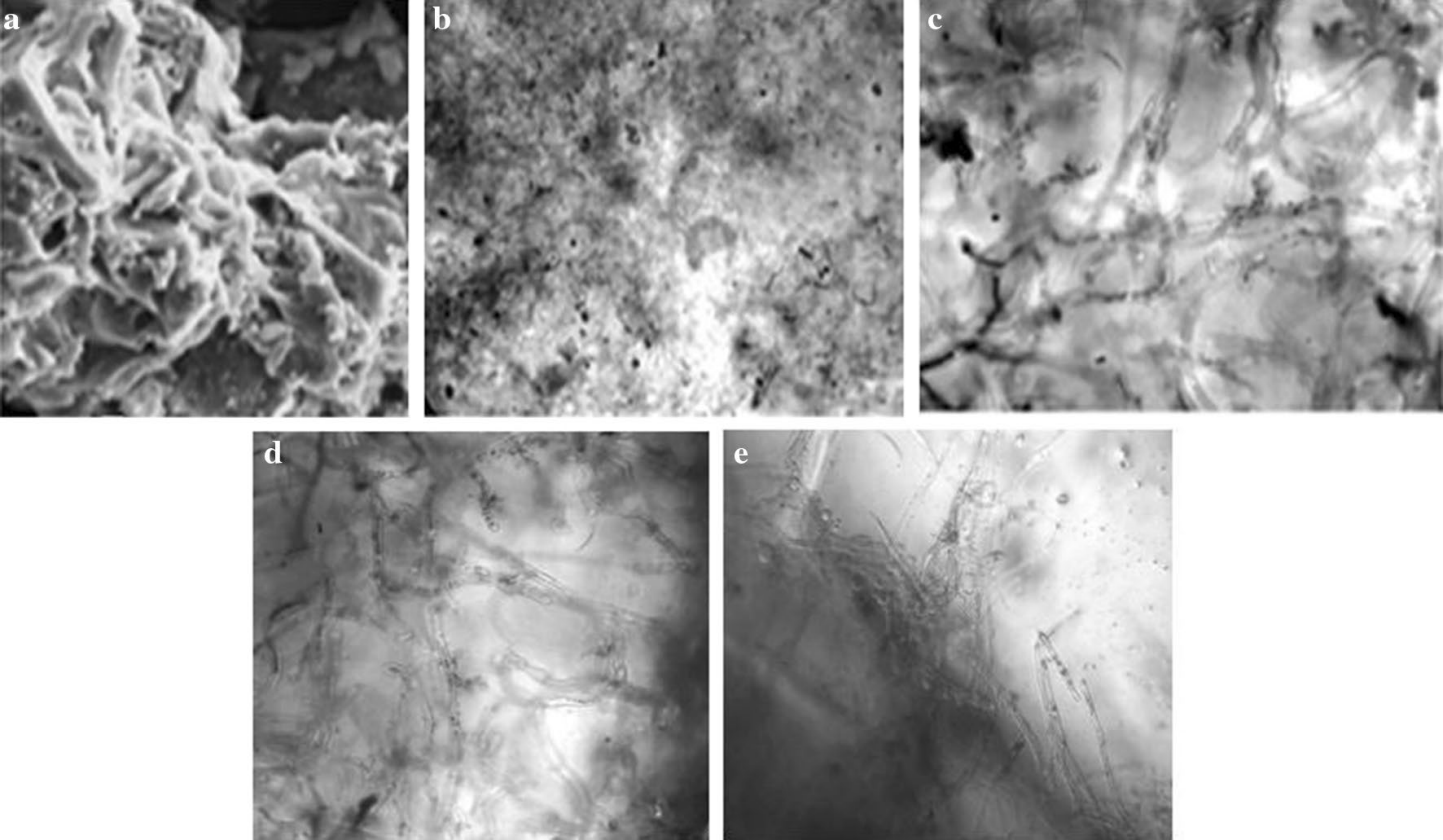
Microscopic view of *S. roseosporus* cells. **a** Refractory brick (SEM), **b** refractory brick, **c** free mycelium **d** silk sachets **e** pellets (cross section)

*Streptomyces roseosporus* cells as free cells and immobilized cells were studied by electron microscopy. The SEM photograph showed that cells were randomly distributed in the pores of the carrier matrix as seen in Fig. 6. The photographs show the inner pores of carriers used, which were densely populated by the immobilized cells. The porous network was covered with the *S. roseosporus* cells which were adsorbed on to the matrix surface. The electron microscopic view of the immobilized cells revealed their dense and compressed structure. The mycelial network was compact and condensed to a smaller area. The silk sachets packaged the mycelium well enough but did not compress them much. The elongated, free mycelium were distributed over the space while the pelletized cells congregated under stress conditions.

### Rheological characterization of the broth

**Fig. 7:**
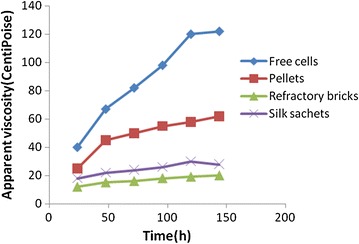
Apparent viscosity of the fermentation broth over the time

Figure 7 shows that morphological changes and rheological properties of *S. roseosporus* were interdependent phenomena. The three phase broth viscosity increased over the time for both free and pelletized cells. The specific cell growth rate was more for the free forms of cells than immobilized cells. The free cells accumulated as dense aggregates and resulted in viscous broth during the course of fermentation. The viscosity increased three times almost the initial value. This majorly affected the oxygen mass transfer. The pellets were comparatively stable but the dense growth of cells over the time led to 1.5 times increased viscosity and reduced mass transfer. The slow and steady growth of immobilized cells helped them overcome the viscosity issue and hence improve the hydrodynamics of the system.

### Variation of volumetric oxygen transfer coefficient

**Fig. 8:**
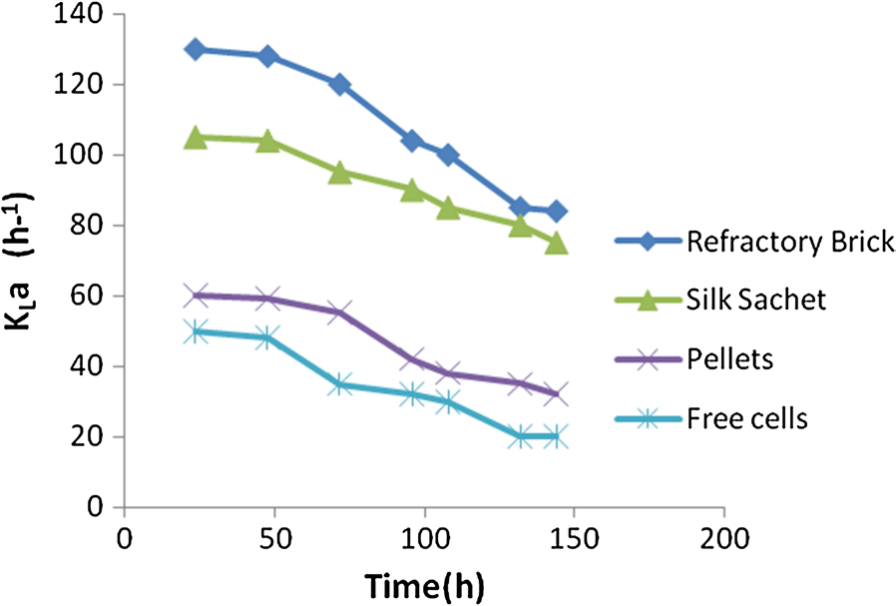
Volumetric oxygen mass transfer pattern for various systems

The variation of volumetric oxygen transfer coefficients by immobilized cells on different adsorbent along with free cells. It was observed that, with free cells, the volumetric oxygen transfer coefficients could be maintained at 20 h^−1^ at 144 h as depicted in Fig. 8. This effectively resulted in reduced Daptomycin. However, with the use of immobilized modes, volumetric oxygen transfer improved as three phase fluid viscosity of the broth was much better controlled.

Volumetric oxygen mass transfer coefficients observed at 132 h were 85 and 80 h^−1^ for refractory bricks and silk respectively whereas pellet was low. Broth viscosity was controlled in case of immobilized cells.

## Discussion

The filamentous nature of actinomycetes leads to its shear-sensitivity. Therefore, airlift bioreactor was chosen. Daptomycin production with free cells, pelletized cells and immobilized cells on various support matrices depicted the correlation of morphological variations with antibiotic production. The production of Daptomycin by immobilized cells was compared with that of two morphological forms of the cells i.e. free and pelletized cells. Two types of Pellet growth occurred in media using peptone as nitrogen source and yeast extract at 4 % inoculum size. Smaller pellets formed by peptone supplemented media with 4 % inoculum were more advantageous for antibiotic production due to their high surface to volume ratio (Nielsen et al. 1995). Faster consumption of peptone as a nitrogen source and low inoculum size led to comparatively slower growth of cells and nitrogen starvation. The cells started utilizing cellular nitrogen leading to stress conditions. *S. roseosporous* is a strict aerobe. It is necessary to provide sufficient dissolved oxygen during the fermentation. The dissolved oxygen tension in bulk fluid was increased by lowering the air flow rate to 0.70 vvm which led to high oxygen tension on the surface and in the centre of the pellets. Previous literature(s) support that improving DO tension in submerged cultivations is favorable for the pellet formation, as described in (Du et al. 2003). The consolidated data in Table 3 shows that in a single batch, pelletized cells and free cells depicted considerable production ability.

**Table 3:** Comparison of Daptomycin production by free and immobilized cells of *S. roseosporus*

Carrier material	Daptomycin conc. (mg/l) (after first batch)	Daptomycin conc. (mg/l) (after final batch)	Reusability up to (cycles)
Pellets	810	1430	2
Free cells	750	750	Nil
Refractory brick	700	4895	8
Silk sachets	620	3623	6

Pellet growth offered advantages over the free cells and the regulation of hyphal extension and pellet size are of great importance. But the reusability and controlled fluid viscosity of immobilized cells had an edge over them. This is beneficial for antibiotic fermentation which is non growth associated (Ramakrishna and Prakasham 1999).The increased production of Daptomycin in immobilized system over free cells is accounted due to changes in permeability of cell walls well as reusability of the matrices (Morikawa et al. 1979). For pelletized cells, the reusability was limited up to two cycles as the cells degenerated, showed dense aggregates and hence increased broth viscosity. 1430 mg/l Daptomycin was produced using pellets for 2 batches. Immobilized cells on refractory bricks and silk sachets led to 4895 mg/l and 3623 mg/l Daptomycin production respectively. The cell leakage, increased viscosity and cell degeneration over the time led to lesser reusability of silk sachets than refractory bricks. The high oxygen demand of the Daptomycin production processes may be attributed to its demand in the biosynthetic pathway. The biosynthetic pathway of Daptomycin shows that there are two crucial oxygen consuming steps in the pathway (Tally and De Bruin 2000; Robbel and Marahiel 2010):

1. The initiation step of Daptomycin formation requires oxygen where lipidation by DptE and DptF takes place. Decanoic acid is activated by the putative adenylating enzyme DptE under ATP consumption. The fatty acid is then transferred onto acyl carrier protein DptF. The C domain of DptA is predicted to catalyze the condensation reaction between fatty acid and *N*-terminal tryptophan.
2. The cyclization and elongation of the peptide chain where an adenylation domain selects the amino acid monomer to be incorporated and activates the carboxylate with ATP to make the aminoacyl-AMP. Next, the A domain installs an aminoacyl group on the thiolate of the adjacent T domain. The condensation (C) domain catalyzes the peptide bond forming reaction, which elicits chain elongation.

The dense network of the free cells led to three phase fluid viscosity which hampered the oxygen mass transfer into the cells. For the immobilized system, the three phase broth viscosity was much better controlled and the hydrodynamics improved over the time. Volumetric oxygen mass transfer coefficients observed at 132 h were 85 h^−1^ and 80 h^−1^ for refractory bricks and silk respectively whereas that for pellet and free cells was low. The present study confirmed that Daptomycin production with whole cell immobilization strategy in an airlift bioreactor showed improved results. Therefore, whole-cell immobilization can be considered as a useful strategy for enhanced production of this life-saving drug. Further, efforts can be taken to improve the mass transfer characteristics of the free cell systems.

## Abbreviations

NBRC: National Biological resource Centre, Chiba, Japan
VVM: volume of air flow per unit of volume of media per minute (volume per volume per minute)
SEM: scanning electron microscopy
